# Detection of Pyrazinamide Heteroresistance in Mycobacterium tuberculosis

**DOI:** 10.1128/AAC.00720-21

**Published:** 2021-08-17

**Authors:** Jim Werngren, Mikael Mansjö, Mikaela Glader, Sven Hoffner, Lina Davies Forsman

**Affiliations:** a Department of Microbiology, The Public Health Agency of Sweden, Stockholm, Sweden; b Department of Global Public Health, Karolinska Institutet, Stockholm, Sweden; c Division of Infectious Diseases, Department of Medicine, Solna, Karolinska Institutet, Stockholm, Sweden; d Department of Infectious Disease, Karolinska University Hospital, Stockholm, Sweden

**Keywords:** BACTEC MGIT 960, *Mycobacterium tuberculosis*, Wayne's test, drug susceptibility testing, heteroresistance, method evaluation, pyrazinamide, whole-genome sequencing

## Abstract

Heteroresistance is defined as the coexistence of both susceptible and resistant bacteria in a bacterial population. Previously published data show that it may occur in 9 to 57% of Mycobacterium tuberculosis isolates for various drugs. Pyrazinamide (PZA) is an important first-line drug used for treatment of both drug-susceptible and PZA-susceptible multidrug-resistant TB. Clinical PZA resistance is defined as a proportion of resistant bacteria in the isolate exceeding 10%, when the drug is no longer considered clinically effective. The ability of traditional drug susceptibility testing techniques to detect PZA heteroresistance has not yet been evaluated. The aim of this study was to compare the capacity of Bactec MGIT 960, Wayne’s test, and whole-genome sequencing (WGS) to detect PZA-resistant subpopulations in bacterial suspensions prepared with different proportions of mutant strains. Both Bactec MGIT 960 and WGS were able to detect the critical level of 10% PZA heteroresistance, whereas Wayne’s test failed to do so, with the latter falsely reporting highly resistant samples as PZA susceptible. Failure to detect drug-resistant subpopulations may lead to inadvertently weak treatment regimens if ineffective drugs are included, with the risk of treatment failure with the selective growth of resistant subpopulations. We need clinical awareness of heteroresistance as well as evaluation of new diagnostic tools for their capacity to detect heteroresistance in TB.

## INTRODUCTION

Heteroresistance of Mycobacterium tuberculosis is defined as the simultaneous presence of susceptible and drug-resistant organisms. There are two mechanisms behind heteroresistance: superinfection (i.e., a mixed infection with different strains) and infection by a polyclonal single strain with differences in at least one nucleotide in a drug resistance-conferring region (1–3). Heteroresistance in tuberculosis (TB) is common, and previous studies have shown occurrence from 9% to 57% heteroresistance for drugs such as rifampin (RIF), isoniazid (INH) and fluoroquinolones (FQ) (1, 3–11), up to 83.9% in multidrug-resistant-TB (MDR-TB) isolates (12). Heteroresistance may be an intermediate stage to full resistance, and missed detection of heteroresistance may lead to treatment failure, as susceptible populations are eliminated, leaving room for resistant populations to thrive (4). Despite the risk of poor treatment outcome, heteroresistance has so far received little attention in the TB field. Moreover, it is not commonly considered when evaluating new methods of drug susceptibility testing (DST), even though it has been clearly shown that different methods to detect resistance to some first-line drugs differ greatly in their sensitivity to detect a drug-resistant subpopulation (10, 11, 13). So far, the few available studies have mainly explored detection of heteroresistance for other first-line drugs, whereas pyrazinamide (PZA) heteroresistance has received less attention.

PZA is an important first-line TB drug, which historically enabled the reduction of treatment length from 9 to 6 months due to its sterilizing activity (14). It is a prodrug requiring activation by the enzyme pyrazinamidase (PZAase), encoded by the *pncA* gene (15).

DST of PZA can be divided into phenotypic (e.g., Bactec MGIT 960 [16] and PZAase/Wayne’s test [17]) and genotypic (e.g., Sanger sequencing [18], NiproLiPA [19], and next-generation sequencing [NGS]) (20, 21) tests. Bactec MGIT 960 is based on the proportional method (22), which detects the critical 1% resistance proportion which has been shown to be predictive of poor treatment outcome for core drugs such as RIF and FQ (23). However, a resistance detection level of 10% is used for PZA DST (24), since an acidic medium is required, which hampers growth. The enzymatic Wayne’s test identifies a functioning PZAase (17) that converts PZA into pyrazinoic acid (POA) under acidic conditions, which is bactericidal to the tubercle bacilli. Sanger sequencing was the first widely used sequencing method but is now being replaced by NGS assays such as whole-genome sequencing (WGS) in many settings (18, 25). WGS has now replaced primary phenotypic DST for all positive mycobacterial cultures in England. As PZA treatment for PZA-susceptible *M. tuberculosis* has been linked to successful treatment outcome (26, 27), it is important not to overlook heteroresistance.

As of today, the accuracy of determining heteroresistance by traditional DST methods of known proportions of heteroresistance has not been reported for PZA. Therefore, we evaluated the abilities of Bactec MGIT 960, Wayne’s test, and WGS to detect the critical level of 10% PZA heteroresistance in experimentally mixed bacterial populations.

## RESULTS

### Bactec MGIT 960 detected heteroresistance in 13 of the 15 mixtures with the 10% PZA resistance proportion.

**FIG 1:**
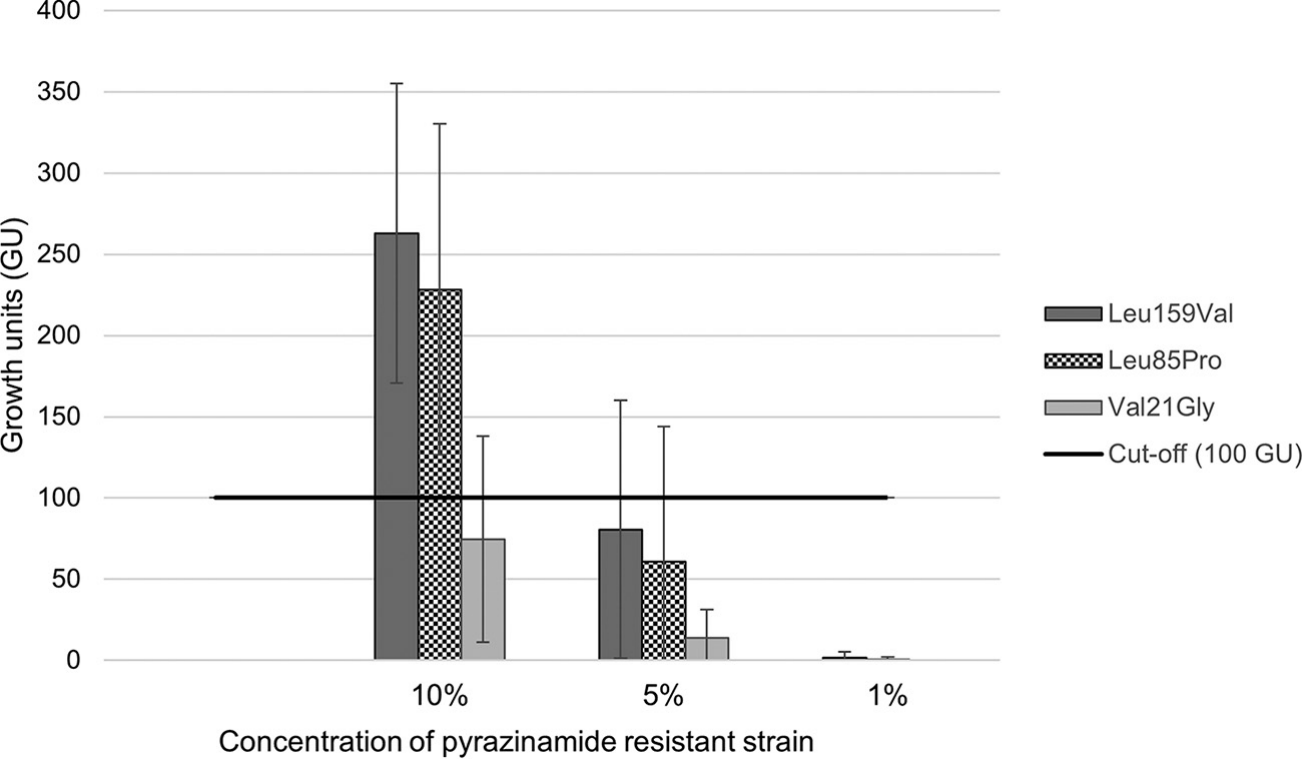
Bactec MGIT 960 results. Histogram showing the mean growth units (GU) with standard deviations for the 10%, 5%, and 1% mixes of the *in vitro*-generated *pncA* Leu159Val and Leu85Pro mutants as well as the *pncA* Val21Gly mutant MDR isolate. A result of ≥100 GU (marked by the horizontal line) indicates pyrazinamide resistance. The tests were performed in replicates on one occasion and in duplicate on two separate occasions, i.e., 5 tests per mixed proportion (10%, 5%, and 1%) of each PZA-resistant strain (*n* = 45 tests in total).

The 10% proportion of PZA-resistant mutants was detected by Bactec MGIT 960 on all but one occasion, where the PZA-resistant clinical isolate (in duplicate) was detected a day after the 1/10 control reached 400 growth units (GU) (Fig. 1). The 1/10-diluted controls reached 400 GU in 5 to 6 days for all tests, indicating that the concentration of the bacterial inoculums was optimally adjusted.

### Wayne’s test was unable to detect PZA heteroresistance—risk of false PZA susceptibility reporting.

**FIG 2:**
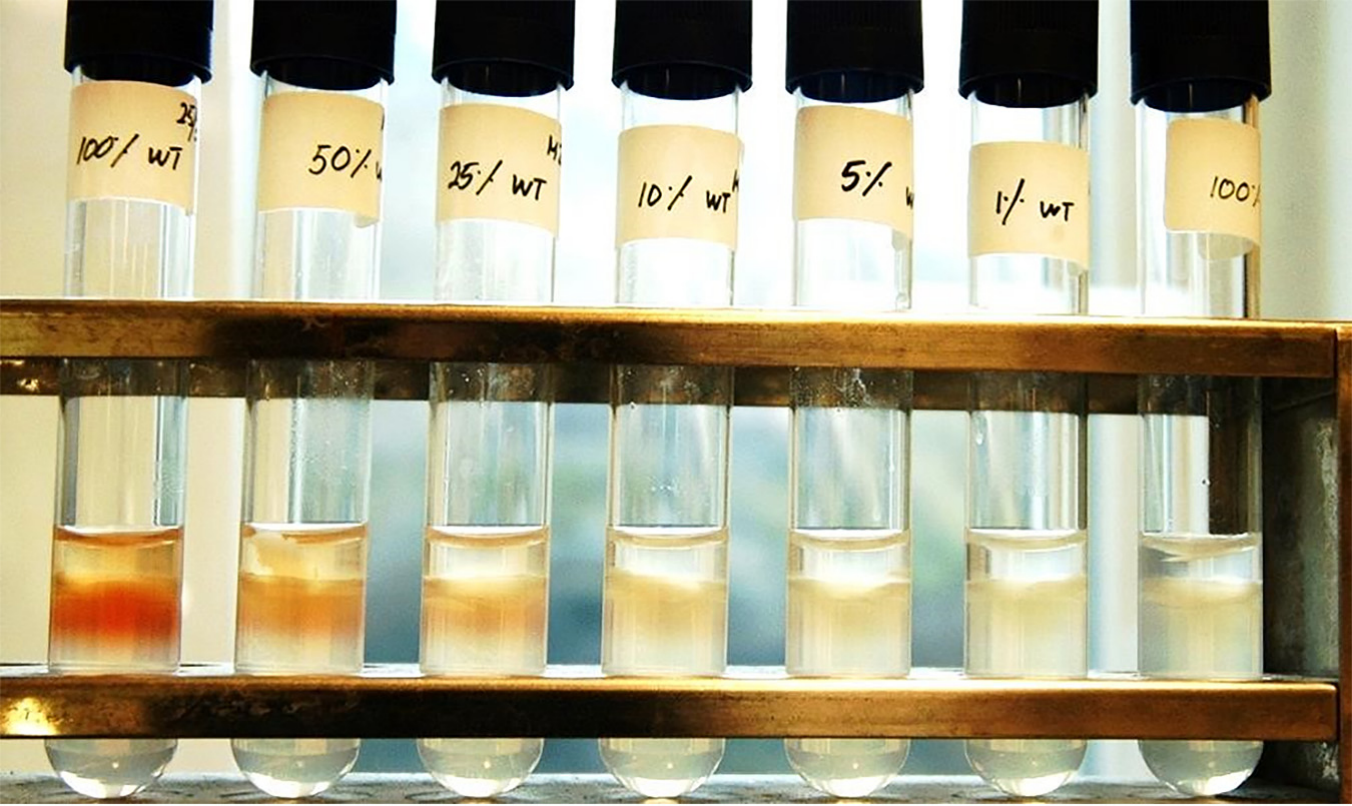
Results of Wayne’s test with the mixes of H37Rv and the *in vitro-*generated isogenic H37Rv PZA-resistant mutant Leu159Val. From the left: 100% susceptible H37Rv and thereafter decreasing proportions of H37Rv (50%, 25%, 10%, 5%, and 1% H37Rv, mixed with the Leu159Val mutant.) A positive sample, i.e., a PZA-susceptible sample, forms a red band below the agar surface. The tubes containing 100% H37Rv and 50% H37Rv are clearly positive, while 25% H37Rv showed a weaker positivity. The remaining samples, i.e., those with 10%, 5%, and 1% H37Rv and with 100% Leu159Val mutant, are negative and regarded as PZA resistant.

Wayne’s test showed positive results, i.e., PZA susceptibility, for 100% H37Rv and for H37Rv mixed with 50% resistant *pncA* mutant. There was a borderline positive result, indicating susceptibility, in samples containing 25% H37Rv, even though they contained 75% PZA-resistant mutants. The mixes with 10%, 5%, and 1% H37Rv were all negative and were interpreted as resistant (Fig. 2). The results were similar for both the isogenic Leu159Val and Leu85Pro mutants.

### WGS detected 10% PZA heteroresistance with good reproducibility.

**FIG 3:**
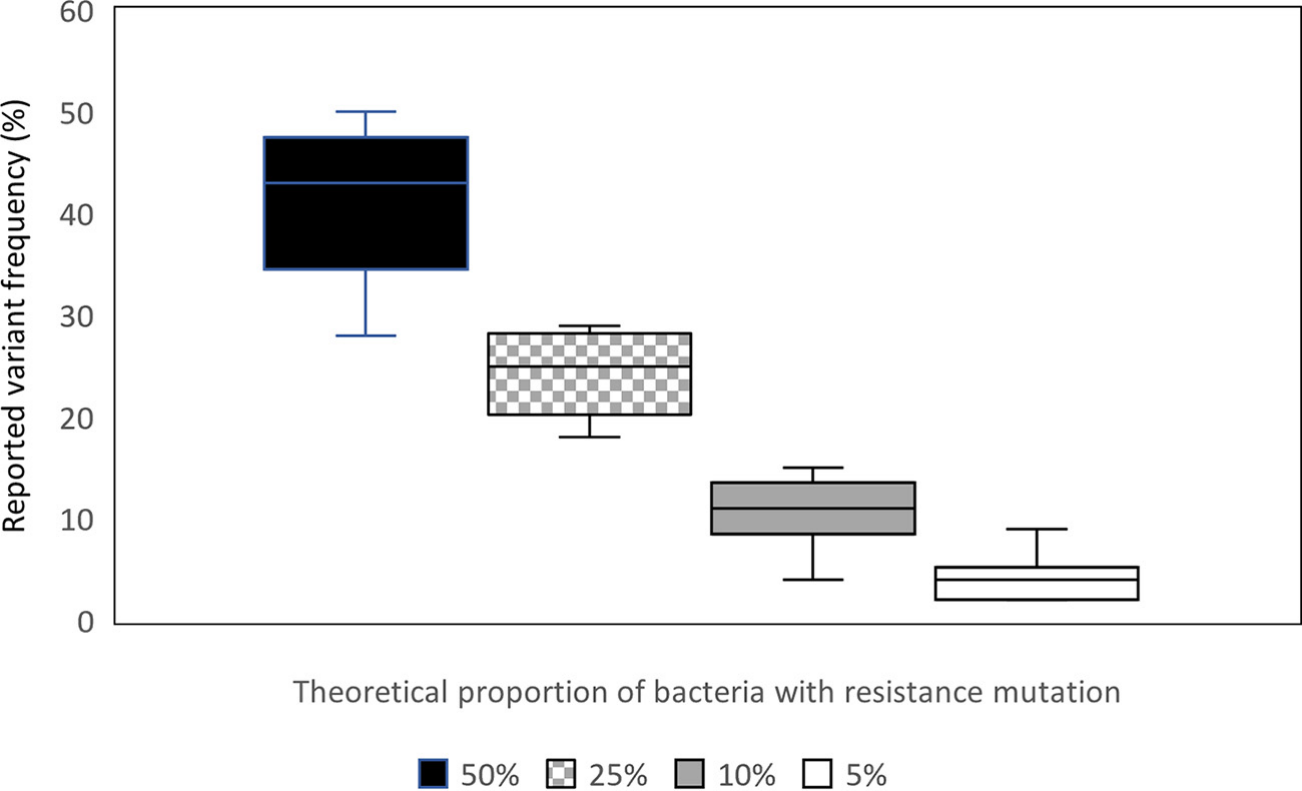
Reproducibility of the whole-genome sequencing (WGS) assay to detect different levels of heteroresistance in cultures with 50, 25, 10, and 5% resistant bacteria (each proportion was tested for six different cultures). The horizontal lines in the boxes represent median values. Three of four mixtures with 5% resistant bacteria were detected only after a less strict variant filtering was applied. Data are based on sequencing of 5 cultures containing 50% resistant bacteria. One of the mixtures with 50% resistant bacteria did not yield enough DNA to be subjected to WGS.

WGS data were successfully obtained from all mixtures except three (insufficient material for sequencing in one of the 50% mixtures and two of the 1% mixtures). By applying the filtering steps described above, all resistant subpopulations were detected in the remaining replicates containing 50%, 25%, and 10% resistant bacteria but in only one of the mixtures containing 5% resistant bacteria (Table S2). In the remaining samples containing 5% resistant bacteria, we managed to identify the expected variants, but only by applying less strict filtering (in these cases, the mutations were detected in only one sequencing read and/or with a forward/reverse balance of 0). Among the four sequenced 1% mixtures, the expected variant was detected in only one case and then by using the less strict filtering parameters. The reproducibility of the assay to detect heteroresistance is shown in Fig. 3.

## DISCUSSION

This is the first study, to our knowledge, evaluating the capacity of Bactec MGIT 960, Wayne’s test, and WGS to detect PZA heteroresistance in experimentally mixed *M. tuberculosis* populations. We found that the phenotypic culture-based (Bactec MGIT) and genotypic (WGS) tests were both able to adequately detect PZA heteroresistance at the critical 10% level, whereas Wayne’s test repeatedly failed in this regard. In addition, a significant risk of false PZA susceptibility reporting with Wayne’s test was seen.

Bactec MGIT 960 is the only DST method of PZA endorsed by the World Health Organization (24), although it is mainly performed in high-resource settings. It requires long incubation times, and resistant populations can be outgrown by susceptible, fitter bacteria, leading to false-negative results (28).

Previous studies support our results where Bactec MGIT 960 succeeded in detecting the critical 1% proportion resistant to both RIF and INH (10, 11). Similarly, for fluoroquinolones, mutations in *gyrA* and *gyrB* present in ≥1% of the population were detected by Bactec MGIT (13).

In two (duplicates) of our 15 Bactec MGIT tests for the detection of 10% PZA heteroresistance, the result of the mixture with the clinical isolates was just below the limit (100 GU) at the time of detection. This might be explained by the PZA-resistant isolate exhibiting an MDR-TB pattern, where the mutations may have resulted in a higher biological cost and slower growth, although there was no obvious difference in growth rates of the controls between the susceptible and the MDR isolate in the Bactec MGIT.

Wayne’s test is a low-cost and easy in-house method to determine PZA susceptibility and is used in various laboratories worldwide. However, there is a risk of a false negative, i.e., a PZA-resistant result, if too small an inoculum is used, especially since results are visually, and thus subjectively, interpreted. Despite experienced personnel and duplicated experiments, samples containing a mix of 75% PZA-resistant and 25% PZA-susceptible *M. tuberculosis* showed a slight color change and were determined to be borderline positive, indicating susceptibility to PZA. This would mean that a 75% resistant sample would be regarded as PZA susceptible, although the drug would be an ineffective treatment option. Although a multidrug regimen is used, the inclusion of an ineffective drug increases the risk of treatment failure and further development of drug resistance.

In order to reduce turnaround time, rapid genotypic tests are desirable. Unfortunately, rapid genotypic tests are in general indiscriminative in detecting heteroresistance. For GeneXpert, the limit of detection of RIF heteroresistance in a study from Botswana was >90% resistance, leading to a problem of false susceptibility results (4). Line probe assays (LPAs), such as MTBDR*sl*, allow rapid detection of resistance (29). The limit of detection of MTBDR*sl* for RIF, INH, and gatifloxacin was consistently ≥5% (10, 11, 13). However, it should be emphasized that heteroresistance detection with GeneXpert or LPAs is possible only for the included regions on the probe, leaving other mutants undetected (13). The PZA-specific LPA, NiproLiPA, has not yet been evaluated for detection of heteroresistance (19).

Therefore, our results are promising, as WGS was able to detect the theoretical threshold of a 10% proportion of resistant bacteria. WGS can reliably detect as little as 5% resistant bacteria, depending on the resistance mutation and choice of method. Resistant subpopulations can also be identified by deep sequencing in samples considered sensitive by phenotypic methods (28). WGS has been used to detect heteroresistance in patients, where prospective use of WGS might have led to changes in the treatment regimen (30, 31).

In previous studies evaluating the detection of 1% heteroresistance of RIF (11) and INH (10) by sequencing, only a level of 50% heteroresistance could be detected for both drugs. In the present study, Sanger sequencing was performed for the same heterogenous mixes but failed to detect the critical level of 10% PZA resistance (data not shown). In comparison with Sanger sequencing, WGS has been found to be superior in detecting heteroresistance, as expected (5, 6).

However, there are some limitations with WGS. The detection of subpopulations at frequencies at 1% might be complicated and too expensive for routine use at present (32). Moreover, the data provided by WGS might be bioinformatically complex (6, 33). PZA resistance also occurs by other mechanisms than single nucleotide polymorphism, such as insertions and deletions, which may be more difficult to detect by current WGS pipelines. In settings where phenotypic DST is being gradually replaced by genotypic DST, it could be problematic to apply the less strict filtering parameters used for detection of the 5% resistant population in this study (this is of particular concern for the drugs where the phenotypic DST detects resistant populations down to 1%). The less strict filtering would likely result in a high level of background noise, with sequencing artifacts being reported as true variants. One way to overcome this challenge is to apply only the “less strict filtering” on genomic positions known to harbor resistance mutations and then continue with a confirmatory phenotypic DST on all isolates for which a low-quality resistance mutation has been reported. Nevertheless, targeted deep sequencing has been suggested as the gold standard to detect heteroresistance (13).

Heteroresistance may have major clinical implications, especially in settings with high prevalence and high infection rates, weak treatment regimens, and/or limited diagnostic tools of TB. The rate at which mixed infection occurs depends on the extent of the spread and diversity of *M. tuberculosis* strains in a community, as there is no known immunity from a single-strain infection (3, 34). Heterogeneity in clonal infections is believed to emerge as a result of selective pressure during weak regimens. A weak regimen may result from a combination of poor drug penetration into tuberculous lesions, poor adherence, poor drug supply/quality, or pharmacokinetic variability (1, 12). High frequencies of heteroresistance have been seen in MDR-TB isolates from countries with high prevalence and subgroups of patients, such as HIV-coinfected and retreatment cases (3, 6, 35), and have been associated with having multiple tubercular lesions (12). In a retrospective study, WGS detected heteroresistance in isolates from 26 of 31 MDR-TB patients to ≥1 drug (12).

Since most countries with high prevalence of TB rely on GeneXpert for diagnosis, the risk is that heteroresistance might be systematically overlooked. A falsely susceptible DST result might lead to weak treatment regimens and unnecessary toxicity due to the use of an ineffective drug. This was highlighted in a study from Botswana where 37 of 370 patients with mixed *M. tuberculosis* infections were seen to have an increased risk of poor treatment outcome (failure, default, or death; adjusted odds ratio [aOR], 6.5; 95% confidence interval [CI], 2.1 to 20.5) (4). As the study mainly included MDR-TB, retreatment and HIV patients, more studies are needed regarding the clinical impact of heteroresistance of TB drugs.

The strengths of our study are that we performed all experiments in duplicate to account for intra- and interassay variability. We also used *in vitro*-made mutants as well as clinical strains. The isogenic mutants were derived from H37Rv to harmonize growth characteristics between the strains. Isogenic mutants are presumably identical to the parent strain, except for the *pncA* mutation, thereby minimizing differences in the characteristics of the parent and mutant strains. As for the clinical strains, two isolates from the same lineage were chosen for the reasons mentioned previously. Three commonly used diagnostic tools were used to evaluate the critical detection level of 10% PZA heteroresistance.

In summary, molecular (WGS) and phenotypic liquid culture-based DST were superior to the enzymatic assay for the detection of PZA-resistant subpopulations, whereas Wayne’s test failed to detect PZA heteroresistance at the critical level of 10%. Heteroresistance is often a disregarded phenomenon in TB and a challenge to diagnose (1). Therefore, the implication of this study and the current body of evidence are that clinicians need to be aware of the risk of heteroresistance and request repeat DST if patients with seemingly drug-susceptible TB are not responding to first-line drug treatment. Discordant results between phenotypic and genotypic DST should be reanalyzed and heteroresistance ruled out as a cause of disagreement. When new methods of DST for TB drugs are evaluated, their ability in detecting drug-resistant subpopulations should be considered, as overlooked heteroresistance may have severe clinical implications. Furthermore, we also suggest that future studies include the use of amplicon-based NGS, in order to improve resolution and lower the limit of detection.

## MATERIALS AND METHODS

Heterogenous mixes of *M. tuberculosis* were made according to the method described by Folkvardsen et al. (10), and tests were performed in all the three methods: Bactec MGIT 960 (63 tests in total), Wayne’s test (28 tests in total), and WGS (30 tests in total).

### Strains.

Five different *M. tuberculosis* strains were used to create mixtures with different proportions of PZA-resistant bacteria; the pansusceptible H37Rv (ATCC 25618) with two isogenic H37Rv *pncA* mutants (Leu159Pro and Leu85Pro), as well as two clinical isolates, a pansusceptible strain and a multidrug-resistant (MDR-TB) strain, classified as lineage 3.1.1 (36). The clinical isolates had known drug susceptibility profiles and were selected from the national *M. tuberculosis* isolate collection at the TB reference laboratory at Public Health Agency of Sweden. Both lineage 3.1.1 isolates had the synonymous Ser65Ser mutation in the *pncA* gene, exhibited resistance to all first-line drugs (including PZA), and exhibited the *pncA* mutation 62T→G/Val21Gly. Pyrazinamide MIC determinations were performed for all the strains using the Bactec MGIT 960 standard protocol for PZA drug susceptibility testing (37). H37Rv and the drug-susceptible clinical isolate had PZA MICs of ≤50 mg/liter, whereas the two isogenic H37Rv *pncA* mutants and the MDR-TB isolate had a PZA MIC of >400 mg/liter (Table S1). All strains showed similar growth rates, reaching 400 GU relative to the 1/10 proportional growth control in 5 to 6 days, irrespective of the drug resistance profile.

### *In vitro* selection of isogenic pyrazinamide-resistant mutants.

Liquid cultures of the H37Rv strain were grown to an optical density (OD) of 0.75 at 580 nm and then cultured in dilution series on plates with Middlebrook 7H10 agar with oleic albumin dextrose catalase (OADC) and PZA at 500 mg/liter (pH 6.0). After 21 to 28 days, colonies of PZA-resistant mutants were selected and subcultured on solid Löwenstein-Jensen (LJ) medium, and their PZA resistance was confirmed in Bactec MGIT 960. The *pncA* gene was sequenced from DNA lysates, and two mutants with the following mutations were selected: 475C→G, resulting in the amino acid substitution Leu159Val, and 254T→C, resulting in Leu85Pro.

### Culturing of isolates.

*M. tuberculosis* H37Rv, the two *M. tuberculosis* *pncA* mutants (Leu159Val and Leu85Pro), and the clinical isolates were cultured on solid LJ medium with glycerol at 37°C for 2 to 3 weeks. Two full 1-μl loops of bacteria of each isolate were transferred to a glass tube containing 3 ml phosphate-buffered saline (PBS) and glass beads. The bacterial suspensions were homogenized using an ultrasound water bath and then left to sediment for 30 min. One milliliter of the suspension’s upper phase was transferred to a new tube, and the turbidity was adjusted to approximately 0.5 McFarland using PBS. The samples were further diluted 1:5, and 0.5 ml of this suspension was used to inoculated tubes containing 7 ml Bactec MGIT PZA broth (Becton Dickinson) (pH 5.9) with 0.8 ml of Bactec MGIT PZA supplement (Becton Dickinson), 0.05% Tween 80, and 100 μl of 8,000 mg/liter PZA, which gave a final concentration of 100 mg/liter (added to only the cultures with PZA-resistant strains). The tubes were incubated in a shaker at 37°C for approximately 2 weeks.

### Bactec MGIT 960.

**FIG 4:**
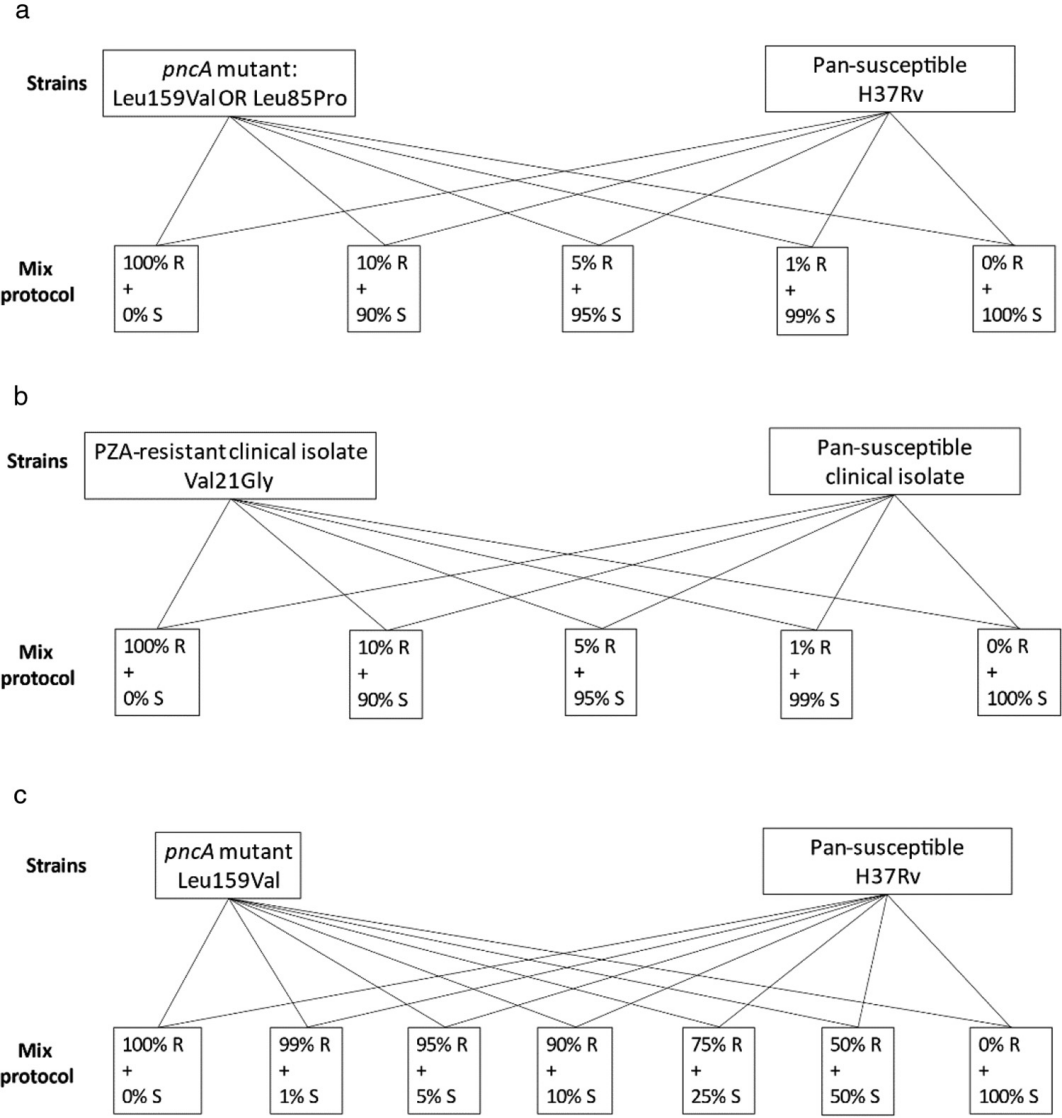
(a and b) Bacterial suspensions prepared with mixed proportions of PZA-resistant *M. tuberculosis* for Bactec MGIT 960. Mixes for whole-genome sequencing were prepared similarly, with the addition of 25% and 50% PZA-resistant bacteria. (a) Isogenic H37Rv mutant and pansusceptible H37Rv. (b) A clinical MDR-TB isolate and a pansusceptible clinical isolate, both lineage 3.1.1. (c) Bacterial suspensions prepared with mixed proportions of PZA-resistant bacteria for Wayne’s test. Similar mixes were prepared for the Leu85Pro mutant.

All cultures were briefly sonicated in an ultrasound water bath to separate potential bacterial aggregates, and OD was measured at 580 nm and adjusted to 0.01 with PBS. Mixed bacterial suspensions were prepared: H37Rv with the *pncA* Leu159Val mutant and H37Rv with the *pncA* Leu85Pro mutant, as well as a mixture of the pansusceptible clinical isolate with the clinical MDR-TB *pncA* Val21Gly isolate (Fig. 4a and b). The mixes contained 0%, 1%, 5%, 10%, and 100% proportions of the PZA-resistant strain (Fig. 4a). A 0.5-ml portion of each mix was inoculated into an MGIT tube with 100 mg/liter PZA. The 1%, 5%, and 10% PZA-resistant mixtures were tested in one replicate on one occasion (*n* = 9 tests) and in duplicate on two separate occasions (*n* = 36 tests) together with a replicate of the 0%- and 100%-resistant suspensions as controls on each test occasion (*n* = 18 tests).

The suspensions were incubated in a Bactec MGIT 960 mycobacterial detection system instrument (Becton Dickinson). When the 1:10-diluted proportional growth control reached 400 GU, the DST was finalized and interpreted. PZA-containing tubes with ≥100 GU were deemed resistant to PZA.

### Wayne’s test.

Wayne’s test is a biochemical colorimetric test for PZA resistance detection (17). In contrast to the detection of PZA-resistant populations in Bactec MGIT and Sanger tests, Wayne’s test detects a functioning PZAase in PZA-susceptible *M. tuberculosis*. Therefore, the bacterial mixtures were reversed for this assay by using low proportions of PZA-susceptible strains, which produce a positive test result, indicated by a red band below the agar medium surface (Fig. 2). Thereby, it was possible to determine at which percentage a PZA-susceptible subpopulation caused a positive result, as an indication of PZA susceptibility. For this test, the cultures of H37Rv and the two *in vitro*-generated *pncA* Leu159Val and Leu85Pro mutants were adjusted to an OD_580_ of 0.2 using PBS, and H37Rv was mixed in 50%, 25%, 10%, 5%, and 1% proportions (including 100% of the H37Rv and the respective mutant as controls) with each of the mutants (Fig. 4c). Tests were performed in replicates at two different occasions (total *n* = 28 tests). No clinical strains were tested using Wayne’s test. The mixtures were centrifuged at 2,900 × *g* for 15 min. Most of the supernatant was discarded, leaving a pellet which was dissolved in the remaining liquid of approximately 0.2 ml. The samples were inoculated in PZAase tubes containing Middlebrook 7H9 agar with glycerol and 400 mg/liter PZA, pH 7 (38). After 7 days of incubation at 37°C, 1 ml of 1% freshly made ferrous ammonium sulfate was added and the results were read visually. Red bands in the agar illustrated a functional PZAase (positive result), indicating the isolate to be susceptible to PZA. If negative, the sample was incubated for an additional 4 h at 4°C before determining the final result.

### Whole-genome sequencing.

Mixtures containing 1% 5%, 10%, 25%, and 50% of the resistant strain were prepared in duplicate as described above (Fig. 4a). The bacteria were subsequently pelleted by centrifugation, and DNA was extracted with QIAamp DNA minikit (Qiagen, Hilden, Germany). WGS (minimum average genome read depth, >20×) was performed on an Ion Torrent platform (Thermo Fisher Scientific, Inc., Waltham, MA) and the obtained sequencing reads were analyzed in CLC Genomics Workbench (version 12.0.3; Qiagen, Hilden, Germany). Briefly, data were mapped against the *pncA* gene derived from the H37Rv reference genome (GenBank accession no. NC_000962.3), and variants were called using the following filtering steps: minimum sequencing depth = 5, minimum frequency of reads calling single nucleotide polymorphisms = 1%; minimum count > 1; forward/reverse balance > 0.

### Data availability.

Raw reads were submitted to the European Nucleotide Archive (accession no. PRJEB44009).
