# Supplementary material for: Detection of Pyrazinamide Heteroresistance in Mycobacterium tuberculosis
Source: Antimicrob Agents Chemother. 2021 Aug 17;65(9):e00720-21. doi: 10.1128/AAC.00720-21 (PMC8370246; doi:10.1128/AAC.00720-21)
Supplement: Supplemental file 1 — Supplemental Tables S1 and S2. Download AAC.00720-21-s0001.pdf, PDF file, 0.5 MB [file aac.00720-21-s0001.pdf]

Supplemental material Table S1. Pyrazinamide (PZA) minimum inhibitory concentrations. The strains were tested at 50-400 mg/L in two-step serial dilutions in BACTEC MGIT 960.

***M. tuberculosis* strain**	**PZA 50 mg/L**	**PZA 100 mg/L**	**PZA 200 mg/L**	**PZA 400 mg/L**
BTB14-036 PZA-S (*pncA*_Ser65Ser)	S	S	S	S
BTB14-054 PZA-R (*pncA*_Val21Gly+Ser65Ser)	R	R	R	R
H37Rv 25618 (pansusceptible/*pncA*_WT)	S	S	S	S
H37Rv iso PZA-R (*pncA*_Leu159Val)	R	R	R	R
H37Rv iso PZA-R (*pncA*_Leu85Pro)	R	R	R	R

Supplemental material Table S2. Whole genome sequencing results and detailed descriptions of the sequenced cultures. Six different cultures were tested.

Sample ID	Description	Proportions	Mean genome coverage	Mean pncA coverage	pncA genotype	Resistant population detected	Comment
A1	Mixtures of H37Rv ATCC 25618 (*pncA*_WT) and its isogenic *pncA* mutant (*pncA*_Leu159Val)	50% Leu159Val/50% WT	36.13	28.79	Leu159Val (28%)	Yes	
A2		25% Leu159Val/75% WT	25.10	24.71	Leu159Val (28%)	Yes	
A3		10% Leu159Val/90% WT	36.03	32.56	Leu159Val (11%)	Yes	
A4		5% Leu159Val/95% WT	42.06	29.32	WT	No*	*Mutation detected at 4%, but only in one read (forward/reverse balance=0)
1		50% Leu159Val/50% WT	56.71	48.13	Leu159Val (43%)	Yes	
B2		25% Leu159Val/75% WT	48.15	43.44	Leu159Val (28%)	Yes	
B3		10% Leu159Val/90% WT	27.11	22.17	Leu159Val (13%)	Yes	
B4		5% Leu159Val/95% WT	59.47	46.72	WT	No*	*Mutation detected at 2%, but only in one read (forward/reverse balance=0).
C1	Mixtures of H37Rv 25618 (*pncA*_WT) and its isogenic *pncA* mutant (*pncA*_Leu85Pro)	50% Leu85Pro/50% WT	44.31	31.03	Leu85Pro (41%)	Yes	
C2		25% Leu85Pro/75% WT	42.92	40.30	Leu85Pro (29%)	Yes	
C3		10% Leu85Pro/90% WT	40.19	38.49	Leu85Pro (15%)	Yes	
C4		5% Leu85Pro/95% WT	53.40	45.56	WT	No*	*Mutation detected at 2%, but only in one read (forward/reverse balance=0).
D1		50% Leu85Pro/50% WT	Not sequenced*	-	-		*Not enough DNA for sequencing.
D2		25% Leu85Pro/75% WT	27.83	24.04	Leu85Pro(21%)	Yes	
D3		10% Leu85Pro/90% WT	24.77	24.04	Leu85Pro(10%)	Yes	
D4		5% Leu85Pro/95% WT	72.82	54.69	Leu85Pro(9%)	Yes	
E1		50% Val21Gly+Ser65Ser/50% Ser65Ser	64.45	58.33	Val21Gly (45%), Ser65Ser	Yes	

E2	Mixture of two clinical lineage 3.1.1 strains BTB 14-036 (PZA-S, *pncA*_Ser65Ser) and BTB 14-054 (PZA-R, *pncA*_Val21Gly+Ser65Ser)	25% Val21Gly+Ser65Ser/75% Ser65Ser	69.47	73.39	Val21Gly (18%), Ser65Ser	Yes	
E3		10% Val21Gly+Ser65Ser/90% Ser65Ser	63.38	50.75	Val21Gly (4%), Ser65Ser	Yes	
E4		5% Val21Gly+Ser65Ser/95% Ser65Ser	71.33	65.43	Ser65Ser	No*	*Val21Gly detected at 2%, but only in one read (forward/reverse balance=0).
F1		50% Val21Gly+Ser65Ser/50% Ser65Ser	48.80	49.40	Val21Gly (50%), Ser65Ser	Yes	
F2		25% Val21Gly+Ser65Ser/75% Ser65Ser	62.02	60.59	Val21Gly (22%), Ser65Ser	Yes	
F3		10% Val21Gly+Ser65Ser/90% Ser65Ser	66.78	62.18	Val21Gly (11%), Ser65Ser	Yes	
F4		5% Val21Gly+Ser65Ser/95% Ser65Ser	64.64	62.98	Ser65Ser	No*	*Val21Gly detected at 4%, but with forward/reverse balance = 0.

*
